# A novel secondary ALK gene mutation which resistant to second-generation TKIs: a case report and literature review

**DOI:** 10.3389/fonc.2024.1430350

**Published:** 2024-08-29

**Authors:** Xiaqin Cheng, Jia Liu, Qiongxia Hu, Yingchun Gao, Lin Zhou

**Affiliations:** ^1^ Thoracic Oncology Ward, Cancer Center, and State Key Laboratory of Biotherapy, West China Hospital, Sichuan University, Chengdu, Sichuan, China; ^2^ Department of Oncology, Chengdu First People’s Hospital, Chengdu, Sichuan, China; ^3^ Department of Precision Medicine Center, West China Hospital, Sichuan University, Chengdu, Sichuan, China; ^4^ Department of Oncology, The People’s Hospital of Pengzhou, Pengzhou, Sichuan, China

**Keywords:** secondary ALK mutation, EML4-ALK gene fusion, second-generation ALK-TKIs, Non-small cell lung cancer, case report

## Abstract

**Background:**

Adenocarcinoma with positive echinoderm microtubule-associated protein-like 4 gene and anaplastic lymphoma kinase (EML4-ALK) gene fusion accounts for 3-7% of lung cancer cases and can be targeted with ALK tyrosine kinase inhibitors (TKIs). Second-generation TKIs are the standard of care for targeted populations, especially those with central nervous system (CNS) metastasis. However, most patients eventually experience disease progression because of drug resistance caused by multiple mechanisms, predominantly secondary mutations.

**Case description:**

We present a female advanced non-small cell lung cancer (NSCLC) case with positive EML4-ALK gene fusion, in which disease progression occurred in only 3 months after first-line treatment with alectinib. Two secondary mutations were detected by next-generation sequencing; one was V1180L located in exon 23, and the other was E803Q located in exon 14, which was a novel mutation that had never been reported. Ensartinib and ceritinib were administered as second-line and third-line treatments. However, the response to these TKIs was poor, and her overall survival was only 7 months.

**Conclusion:**

The secondary mutation E803Q located in exon 14 seems resistant to most second-generation ALK-TKIs. If there is an opportunity, the efficacy of the third-generation ALK-TKI loratinib should be tested.

## Introduction

Adenocarcinoma with positive anaplastic lymphoma kinase (ALK) rearrangement accounts for 3-7% of lung cancer cases (1). Although it is responsible for a low proportion of non-small cell lung cancer (NSCLC) cases, the actual number of ALK-positive NSCLC patients is great due to its high prevalence worldwide. The ALK protein is a transmembrane receptor that belongs to the insulin superfamily and can be activated by gene fusion, resulting in uncontrolled downstream signal activation and promoting tumorigenesis (2, 3). To date, more than 20 types of fusion partners have been described in numerous malignancies, while echinoderm microtubule-associated protein-like 4 gene and anaplastic lymphoma kinase (EML4-ALK) variants are the most common translocations in NSCLC (4). This translocation can be effectively targeted by tyrosine kinase inhibitors (TKIs), which has dramatically changed the therapeutic landscape of lung cancer. The second-generation ALK-TKI alectinib has been approved as a first-line treatment for NSCLC patients with ALK rearrangement according to its low toxicity and promising antitumor activity, especially for brain metastases (5). However, despite this successful advance, most patients will inevitably experience disease progression because of drug resistance from multiple mechanisms, one of which is secondary mutation. Here, we report an advanced lung adenocarcinoma case with positive ALK rearrangement and a novel secondary mutation and its response to second-generation ALK-TKIs.

## Case report

**Figure 1:**
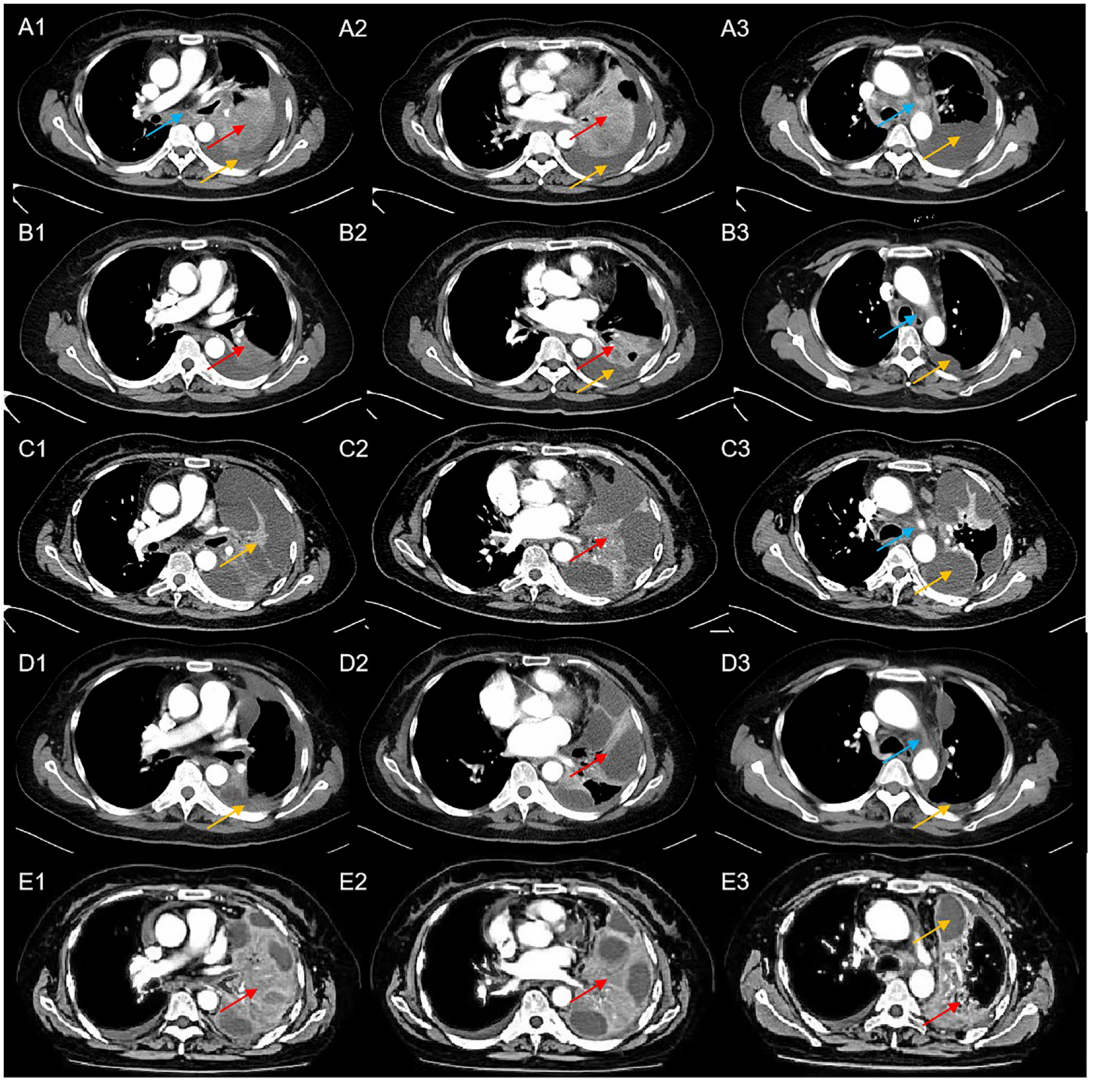
Computed tomography scans of the patient at a particular course of disease (blue arrows indicated mediastinal lymphadenopathy, red arrows indicate the location of the tumor, yellow arrows indicated left pleural effusion); **(A1-A3)** CT scans at baseline; **(B1-B3)** CT scans after 1 month of treatment with alectinib revealed a dramatic reduction in tumor size and pleural effusion; **(C1-C3)** CT scans after three months of treatment with alectinib showed disease progression; **(D1-D3)** CT scans after one month of treatment with ensartinib revealed partial response of the primary tumor and pleural effusion; **(E1-E3)** CT scans after two months of treatment with ensartinib revealed disease progression.

**Figure 2:**
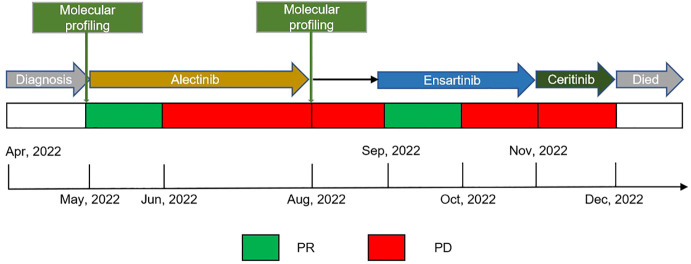
Treatment timeline, the treatment schedule showed the administration of multiple therapeutic TKIs and associated clinical response or outcomes; Black arrow indicated patient continuing on alectinib at the time of waiting for results of molecular profiling. TKIs, tyrosine kinase inhibitors; PR, partial response; PD, progressive disease.

In April 2022, a 56-year-old Asian woman with a smoking history of 20 pack years and no significant family history complained of a dry cough lasting over one month that progressed to dyspnea and hemoptysis after antibiotic treatment. A large mass in the lower lobe of the left lung, ipsilateral atelectasis caused by left main bronchi obstruction, mediastinal and left hilum lymphadenopathy, and left pleural effusion were revealed by contrast-enhanced thoracic computed tomography (CT) (**Figures 1A1–A3**). Subsequent biopsy performed by bronchoscope and samples of neoplasm from the left inferior lobar bronchus were confirmed to be adenocarcinoma. EML4-ALK rearrangement was detected from the same sample by next generation sequencing (NGS) afterward. No other metastases were found from imaging examinations, and the patient was diagnosed at stage cT4N2M1a (stage IV). Oral alectinib 600 mg twice daily was taken as the first-line treatment for this patient beginning in May 2022. After 1 month of therapy (June 2022), the patient’s symptoms of cough and dyspnea improved significantly, and a partial response was observed in the primary tumor and lymph nodes (**Figures 1B1–B3**). However, only 2 months later, the patient’s symptoms of cough and dyspnea worsened again and were accompanied by left chest pain. Progressed primary tumor, lymph nodes, and pleural effusion were observed by means of imaging tests (**Figures 1C1–C3**). NGS detection for pleura effusion was performed, and a high mutation abundance of EML4-ALK accompanied by two other secondary mutations, V1180L located in exon 23 and E803Q located in exon 14, was found. Consequently, this patient received ensartinib at a dose of 225 mg once daily as the second-line treatment starting in August 2023. Once again, the response to ensartinib could only last for a very limited time. Even though partial response was achieved one month after ensartinib treatment (**Figures 1D1–D3**), local regional disease progression accompanied by unbearable symptoms occurred only one month later (**Figures 1E1–E3**). However, this patient refused to undergo further NGS testing and sequential chemotherapy. Ceritinib was administered at a dose of 450 mg once daily as third-line treatment from Nov. 2023, and it seemed to have no efficacy because this patient succumbed to cancer only one month later in Dec. 2022. The entire treatment timelines were provided in **Figure 2**.

## Discussion

ALK gene rearrangements have been considered one of most frequently dominant oncogenic drivers in lung adenocarcinoma, and matched TKI drugs are recommended as the standard option due to their significant impacts. Alectinib was approved as the first-line treatment for ALK gene rearrangement NSCLC because of the significantly prolonged PFS compared with crizotinib (34.8 months vs. 10.9 months, P<0.0001) (6). For patients who relapsed from the first-line treatment of second-generation ALK-TKIs, the alteration to another second-generation TKI treatment is one potential option. The previously reported case showed that ensartinib achieved encouraging benefits with a PFS of 15 months for patients who relapsed from alectinib, and ceritinib achieved a promising overall survival of 4 years after alectinib-refractory (7, 8) However, for the present case, the PFS for alectinib, ensartinib, and ceritinib treatments were obviously inferior to other reports.

Emerging data shows that more than 20 variants of the EML4-ALK gene fusion have been identified (8), and the most common variants are located in exon 13 of EML4 fused to exon 20 of ALK (V1, E13:A20) or exon 6a/b of EML4 fused to exon 20 of ALK (V3, E6:A20). In the present case, the gene fusion was identified to be V3. In a study reported by Jessica Ja et al., drug resistance mutations of the ALK gene were more routinely observed in V3 than in V11 (9). From the Alex trial, the clinical responses of V1 and V3 to alectinib suggested no significant difference in PFS, OS, objective response rate, and duration of response (5, 9). However, ensartinib is reported to be more active against V1 than V3, exhibiting median PFSs of V1 and V3 of 8.2 months and 1.9 months, respectively (10). This might partly explain the poor efficacy of ensartinib in the present case.

It has been reported that drug resistance due to secondary mutations is identified in approximately one-third of patients treated with ALK TKIs (8). Complex mechanisms of drug resistance have been explored and can be classified into 2 categories: ALK-dependent mechanisms, including secondary mutation and amplification of the ALK gene, and ALK-independent mechanisms, including upregulation of bypass signaling pathways or lineage change (11). Each TKI is associated with a unique mutation spectrum, while the three most common ALK mutations associated with alectinib are G1202R, I1171T/S, and V1180L (11, 12). Our case showed two secondary mutations, V1180L and E803Q, after disease progression from alectinib treatment. Johannes et al. reported that 53% of patients undergo secondary mutations after alectinib resistance, and 43% of patients have between 2 and 5 mutation sites (13). V1180L mutation has been reported as a common mutation site for alectinib by previous preclinical models and clinical studies (13–15) and other reported cases showed a similar poor clinical response of patients with V1180L secondary mutations from alectinib treatment (13). To the best of our knowledge, E803Q is a novel secondary mutation that has never been reported before.

As shown in the present case, ensartinib achieved PR as a second-line treatment, which indicated that the V1180L mutation remained sensitive to ensartinib. From the report from Leora Horn et al, the IC50 of cell lines with the V1180L mutation to ensartinib was only 10 nm, indicating an effective antitumor response (10). However, for the present case, the response to ensartinib only lasted for one month, which indicated that E803Q might be resistant to ensartinib. Similarly, for the case reported by Wang et al, ensartinib only achieved SD for patients who relapsed from alectinb treatment with multiple secondary mutations, including L1196M, G1269A, F1174L, and V1180L, and the PFS for ensartinib was only four monthss (16). It has been demonstrated that the V1180L mutation is still sensitive to ceritinib in preclinical studies (10, 15). However, the clinical impact was not as expected in the present case, and the patient succumbed to cancer only one month after ceritinib treatment. This might be because cancer cells harboring V1180L have already been eliminated by ensartinib, and cancer cells harboring E803Q are resistant to ceritinib.

The ALK protein is a transmembrane tyrosine kinase receptor containing the extracellular region (ECR, residues 1-1038), a single transmembrane helix, and the intracellular region (residues 1116-1620) containing ALK kinase domain (residues 1116-1392). The kinase domain is the main functional region in which the most common secondary resistance mutations, including G1220R, V1180L and I1171T/S, were detected. Indeed, the secondary mutation of E803Q, which was reported in our case, was located in the ECR but not the kinase domain. However, according to the report from Reshetnyak AV et al., the ALK ECR also has a glycine-rich region located at residues 648-1,030, which can continuously activate the descending signaling pathway when binding to associated ligands and forming a stable receptor−ligand complex. They also found that ALK could be more easily phosphorylated through mutations, such as E978A and Y996A, in this region (2). Wang Y-W et al. also reported that the H694R mutation, which is located in the ECR, could promote ALK protein phosphorylation (17). Thus, we infer that the E803Q mutation, which is located in the ECR, may cause drug resistance in a similar way. Furthermore, Murugan AK et al. reported that the K1062M mutation, which is located in the transmembrane helix, has oncogenic functions in cell invasion and growth (18). We suppose that second mutations located outside of tyrosine kinase domain of ALK may also contribute to drug resistance.

There were some limitations of the present case that need to be addressed. The first, lorlatinib and bragatinib were also sequential options after other second-generation ALK-TKI refractoriness (10). However, because lorlatinib and bragatinib were not available in China at that time, the present case had not received these TKIs, and the sensitivity to the E803Q mutation was unknown. The second, no gene mutation tests were performed after relapse of ensartinib and ceritinib treatment, and it was not clear whether the new mutation site affected subsequent treatment or whether only the E803Q mutation was detected. The third, no preclinical experiments were performed to validate the drug resistance of E803Q mutation, and this will be our future work.

## Conclusions

We present a case harboring a novel secondary mutation of E803Q after relapse from alectinib treatment, which enriches the mutation spectrum of second-generation ALK-TKI resistance. Furthermore, the E803Q mutation might be resistant to other second-generation ALK-TKIs, such as ensartinib and ceritinib.

## Data Availability

The raw data supporting the conclusions of this article will be made available by the authors, without undue reservation.
